# Aberrant neurophysiological signaling associated with speech impairments in Parkinson’s disease

**DOI:** 10.1038/s41531-023-00495-z

**Published:** 2023-04-14

**Authors:** Alex I. Wiesman, Peter W. Donhauser, Clotilde Degroot, Sabrina Diab, Shanna Kousaie, Edward A. Fon, Denise Klein, Sylvain Baillet, PREVENT-AD Research Group, Sylvia Villeneuve, Quebec Parkinson Network

**Affiliations:** 1 Montreal Neurological Institute, McGill University, 3801 Rue University, Montreal, QC Canada; 2 Ernst Strüngmann Institute for Neuroscience, Frankfurt, Germany; 3 Department of Psychology, Université du Québec à Montréal, Montréal, QC Canada; 4 School of Psychology, University of Ottawa, Ottawa, ON Canada; 5 Center for Research on Brain, Language and Music, McGill University, Montreal, QC Canada; 6 Douglas Mental Health University Institute, 6875 Bd LaSalle, Verdun, QC Canada

**Keywords:** Parkinson's disease, Parkinson's disease

## Abstract

Difficulty producing intelligible speech is a debilitating symptom of Parkinson’s disease (PD). Yet, both the robust evaluation of speech impairments and the identification of the affected brain systems are challenging. Using task-free magnetoencephalography, we examine the spectral and spatial definitions of the functional neuropathology underlying reduced speech quality in patients with PD using a new approach to characterize speech impairments and a novel brain-imaging marker. We found that the interactive scoring of speech impairments in PD (*N* = 59) is reliable across non-expert raters, and better related to the hallmark motor and cognitive impairments of PD than automatically-extracted acoustical features. By relating these speech impairment ratings to neurophysiological deviations from healthy adults (*N* = 65), we show that articulation impairments in patients with PD are associated with aberrant activity in the left inferior frontal cortex, and that functional connectivity of this region with somatomotor cortices mediates the influence of cognitive decline on speech deficits.

## Introduction

Parkinson’s disease (PD) is the second most common neurodegenerative disorder worldwide^1^, and is characterized by progressive declines in motor function and cognition. Difficulties producing intelligible speech are some of the earliest^2–4^ and most debilitating^5,6^ impairments of PD. Speech production is inherently complex, and speech symptoms in patients with PD are multidimensional^7^; they commonly include hoarse *voice*, imprecise *articulation*, and monotonous *prosody*^4,8^. Alongside reductions in speech volume i.e., hypophonia^9,10^, these impairments are classically termed *hypokinetic dysarthria*^11–14^. However, the best approach to quantifying pathological changes in speech remains unclear. On the one hand, evaluation of dysarthric symptoms by a certified speech-language pathologist using clinical scales is time-consuming but often regarded as the gold standard. On the other, more rapid and inexpensive assessments based on automatically-extracted acoustical measures^4,15,16^ are promising, but may not provide a sufficiently nuanced representation of the disease symptomatology. The optimal outcome of remediation speech therapies in PD is improved intelligibility to human listeners, hence the value of human assessment of speech impairments as an alternative. Human ratings of speech impairments in PD are highly reliable across raters^17–19^, time points^20^, and levels of rater expertise^19^, and can contribute to predicting disease progression^18^ and therapeutic outcomes^20^ better than simpler acoustical metrics.

Beyond the robust quantification of speech impairments in PD, their neurophysiological origins are also unknown. In the healthy brain, speech production engages a distributed and predominantly left-lateralized ensemble of cortical regions including the primary motor, primary auditory, pre-motor, posterior parietal and inferior frontal cortices^21–27^. Temporally, cued speech production requires the integration of incoming phonological information in the left superior temporal cortex, followed by engagement of left inferior frontal cortex (LIFC) for syllabification and temporal ordering of speech, which then overlaps with sustained motor processing in the precentral gyrus and voluntary control of motor initiation in the left middle and superior frontal cortices, and finally monitoring of auditory feedback in left superior temporal regions^24,28–36^. The spectral components of neurophysiological activity related to speech production have also been studied extensively^31,37–55^. They include a key role for alpha (~7–13 Hz) and beta (~15–30 Hz) frequency bands in speech-network regions, and slower delta band (~2–4 Hz) activity in prefrontal regions, for effective production of speech^44–50^^,52^. Interregional beta-band functional connectivity between prefrontal, auditory, and motor cortices is also essential for healthy speech production^44,56^.

Functional neuroimaging studies of patients with PD have shown that speech production recruits greater cerebral blood flow and oxygenation across prefrontal, auditory, and motor regions^57–60^ than in healthy controls, and that this hypermetabolism is normalized by common PD therapies^57,58^. Inter-regional connectivity of the speech circuit also appears to impact speech production in PD^61,62^, with opposing effects of decreased versus increased functional connectivity during speech preparation and production, respectively^63^. The neurophysiological spectrum of these effects is less studied; a limited literature suggests decreased beta oscillations in the subthalamic nucleus^64^ and primary motor cortex^65^ in patients with PD during active speech. Importantly, it remains relatively unclear to what extent these patterns of aberrant neural activity during active speech production under highly controlled experimental conditions relate to the real-world difficulties experienced by patients. Given the key role of rhythmic neural activity in motor and cognitive impairments in patients with PD^66–68^, and the proven and future potential for therapeutic interventions based on frequency-specific neurostimulation in this population^69–71^, a clearer understanding of the spatio-spectral neural bases of speech intelligibility deficits in PD is essential.

In this study, we examine the spectral and spatial definitions of the functional neuropathology underlying reduced speech quality in patients with PD (*N* = 59). Towards this goal, we first quantified patient speech impairments with a novel interactive tool designed for non-specialists – an approach intended to better capture the difficulties with speech intelligibility faced by patients with PD. We then introduce a new brain mapping technique of spectral neurophysiological deviations (the Spectral Deviation Index; SDI) between each patient and a group of demographically-matched healthy controls (*N* = 65). This new metric allows for anatomically-resolved mapping of neurophysiological aberrations in patient participants, without requiring specific hypotheses regarding the expected frequency bands involved. We hypothesized that aberrant neurophysiological manifestations would involve multiple frequency bands, map to brain regions known for their involvement in speech production, and scale with the severity of speech impairments in patients with PD. Based on previous literature indicating beta-frequency connectivity changes in patients with PD^72,73^ and the importance of such connectivity in healthy speech production^44,56^, we also anticipated that neurophysiological beta connectivity across the brain circuit for speech production would be altered in those patients with more pronounced speech difficulties.

## Results

### Demographics, clinical assessments, and speech impairments in patients with PD

**Fig. 1:**
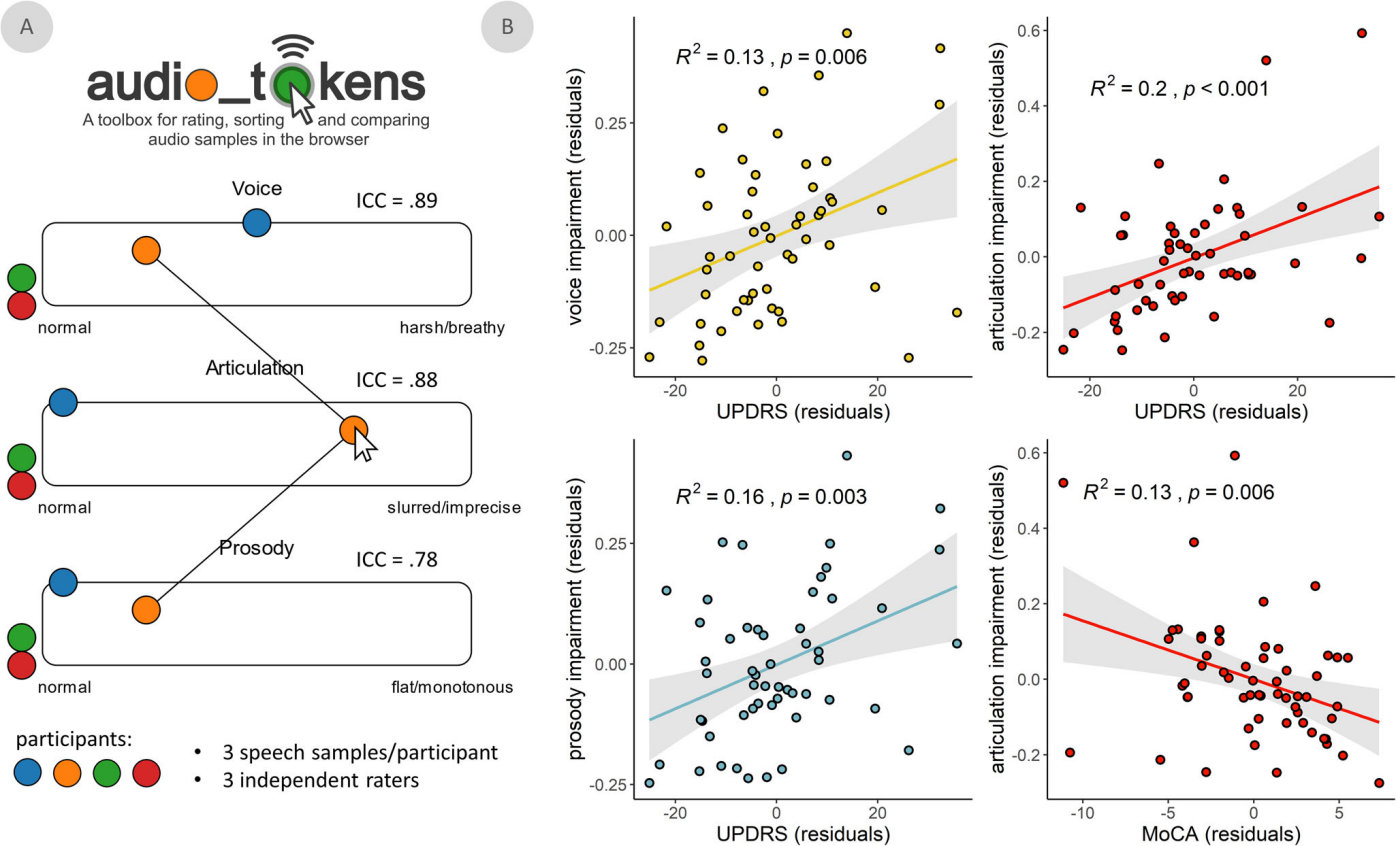
Speech impairment ratings are reliable and associated with clinical features of PD. **A** Graphical user interface for the manual rating of speech feature impairments using *audio_tokens*. When hovered-over with the mouse, colored circles play speech samples from separate patients with Parkinson’s disease (each represented by a different color), allowing for interactive and comparative rating (i.e., by clicking and sliding the circles horizontally) of impairments in each speech feature. Intraclass correlation coefficients (ICC) indicate reliability of speech ratings across three independent raters. **B** Significant linear relationships between voice, articulation, and prosody impairments and two common clinical scales in Parkinson’s disease: the Unified Parkinson’s Disease Rating Scale part III (i.e., UPDRS-III, minus speech sub-scores) and the Montreal Cognitive Assessment (MoCA). All models controlled for age. Shaded intervals represent the 95% confidence interval.

Demographics for both groups, as well as clinical features for the PD group, are reported in Table 1. We used a novel approach to quantify speech intelligibility along three features (i.e., voice, articulation, and prosody) as implemented in the *audio_tokens* toolbox (Fig. 1A)^74^. Speech quality assessments were consistent across raters for all assessed features: voice (intra-class correlation coefficient [ICC] = 0.89, 95% CI = [0.83 0.93]), articulation (ICC = 0.88, 95% CI = [0.81 0.92]), and prosody (ICC = 0.78, 95% CI = [0.66 0.86]; Fig. 1A). These speech impairment ratings were also related to clinical features of PD (Fig. 1B): they were all significantly related to clinical motor function (UPDRS-III, minus speech sub-scores; voice: *t*(51) = 2.82, *p* = 0.007; articulation: *t*(51) = 3.61, *p* < 0.001; prosody: *t*(51) = 3.07, *p* = 0.003), such that greater non-speech motor impairment was associated with greater speech difficulties. We found weaker associations between all three speech features and PD staging (Hoehn & Yahr scale; voice: *t*(45) = 2.05, *p* = 0.046; articulation: *t*(45) = 2.07, *p* = 0.044; prosody: *t*(45) = 2.05, *p* = 0.046; all uncorrected for multiple comparisons). Only articulation impairments were associated with patient cognitive abilities (MoCA scores; voice: *t*(56) = −0.47, *p* = 0.639; articulation: *t*(51) = −2.85, *p* = 0.006; prosody: *t*(51) = −1.15, *p* = 0.250), such that greater cognitive impairment was associated with greater articulation difficulties. Further, these speech ratings related to non-speech motor dysfunction (ΔAIC = -6.69) and cognitive function (ΔAIC = −9.03) better than acoustical features extracted automatically from the same recordings. All three features were also significantly related to speech impairment ratings made by a trained clinician administrator (UPDRS-III speech sub-score; voice: *t*(51) = 5.17, *p* < 0.001; articulation: *t*(51) = 3.51, *p* < 0.001; prosody: *t*(51) = 4.49, *p* < 0.001; Supplementary Fig. 1).

**Table 1:** Between-group demographic comparisons and patient group clinical profile.

	Age (years)	Sex (% male)	Handedness (# left/ambi)	Education (years)
HC	63.02 (8.13)	65	3/3	15.85
PD	65.95 (8.14)	71	4/3	15.14
*p*	0.067	0.434	0.863	0.615

	MoCA	UPDRS-III	UPDRS-III Speech (*N* = 54)	Hoehn & Yahr (*N* = 48)	Years Since Dx (*N* = 56)	% Taking DA Agonists (*N* = 53)
PD						
Range	12–30	7–71	0–3	1–3	1–25	-
Mean (SD)	24.44 (3.99)	32.68 (14.77)	1.35 (0.83)	1.96 (0.70)	6.66 (4.74)	28.81

*HC* healthy control group, *PD* Parkinson’s disease group, *MoCA* Montreal Cognitive Assessment, *UPDRS-III* Unified Parkinson’s Disease Rating Scale part III, *Dx* Diagnosis, *DA* Dopamine. Unless otherwise indicated, values indicate means and associated parentheticals indicate standard deviations. HC sample *N* = 65, PD sample *N* = 59, unless otherwise indicated. *P*-values indicate significance of between-groups comparisons, using Mann–Whitney *U* tests and chi-square tests for continuous and categorical variables, respectively.

### Spectral pathology is related to articulation impairments in PD

**Fig. 2:**
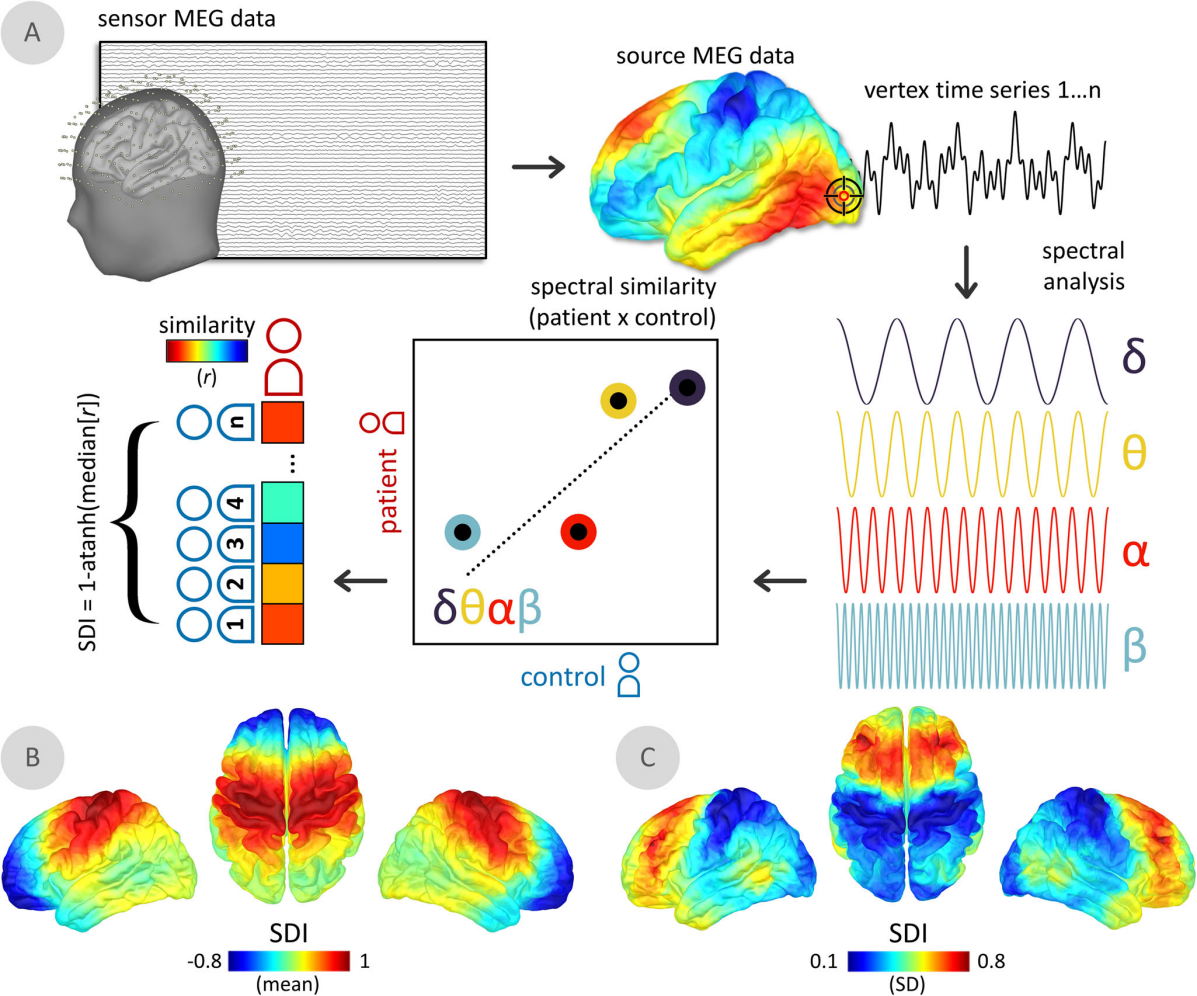
The spectral deviation index (SDI). **A** For all participants, magnetoencephalography (MEG) data were cortically mapped, frequency-transformed, and averaged over canonical frequency bands (i.e., δ/delta: 2–4 Hz; θ/theta: 4–7 Hz; α/alpha: 8–12 Hz; β/beta: 15–29 Hz). Pearson correlation coefficients were then estimated at each vertex, representing the similarity across frequency-wise power estimates between each patient and control. For each patient, the median of these values was taken over all comparisons to control participants, which was then Fisher-transformed to ensure linear scaling (i.e., transformed with the inverse hyperbolic tangent function) and subtracted from 1 to indicate relative deviations from the control group. Surface maps below indicate the mean (**B**) and standard deviation (**C**) of the spectral deviation maps across all patients with Parkinson’s disease.

To examine multi-frequency patterns of neurophysiological change in patients with PD, we developed a metric of absolute (i.e., sign-invariant) spectral deviation across four canonical frequency bands (i.e., the Spectral Deviation Index [SDI]; Fig. 2A). The average of individual SDI maps across all patients emphasized spectral deviations in premotor, primary somatomotor, and superior parietal cortices bilaterally (Fig. 2B). These findings are robust against the binning density of the frequency spectrum (Supplementary Fig. 2A). The greatest variability of SDI across patients was found in the bilateral prefrontal and temporal cortices (Fig. 2C). In most brain regions, the association of the SDI with clinical scores (i.e., MoCA and UPDRS-III) was stronger than that of a linear model parameterized with the four band-limited estimates of neurophysiological power (Supplementary Fig. 3).

**Fig. 3:**
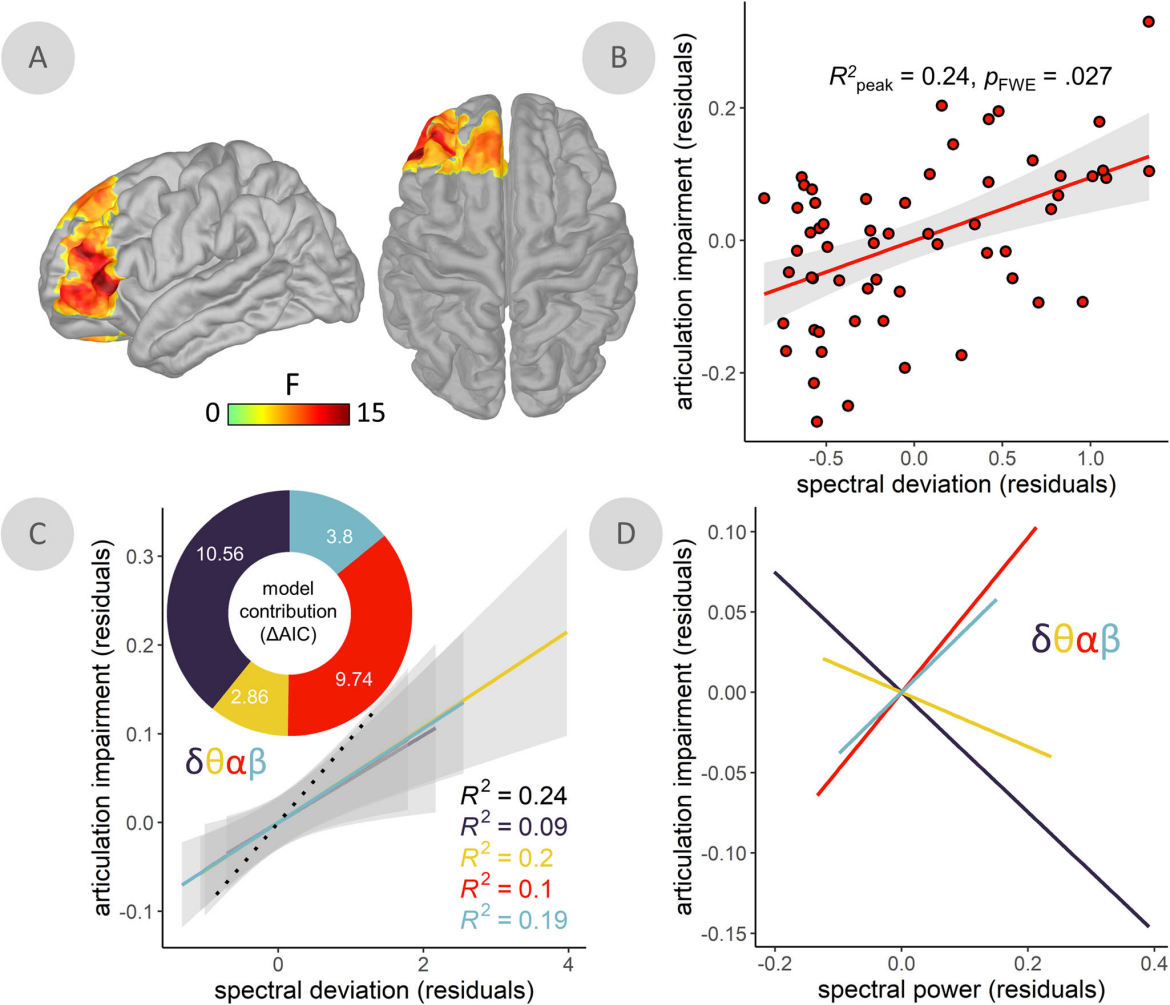
Spectral deviation relates to articulatory impairments in the left inferior frontal cortex. **A** Surface maps indicate a significant relationship between spectral deviations and articulation impairments in patients with Parkinson’s disease (PD), with spectral deviations (SDI) and articulation impairment ratings from the peak vertex of this relationship plotted in (**B**). **C** Model contribution values shown in the donut plot indicate comparisons between models predicting articulation impairment ratings using the SDI values from the same peak vertex and comparable SDI values computed with each frequency left-out once. More positive differences in Akaike information criterion (ΔAIC) indicate a stronger contribution of that frequency to the overall model, with ΔAIC > 2 signifying meaningful differences in model information. Best fit lines represent the linear relationships between these leave-one-out models for each frequency and the articulation impairment ratings across patients with PD (dotted black line = full-frequency, broad-band model). **D** Lines-of-best-fit indicate the direction of the underlying relationships between spectral power at each frequency and articulation impairment ratings. All models controlled for age. Shaded intervals represent the 95% confidence interval.

We performed a multiple regression analysis of these maps on all three speech features, and found that articulation impairments were uniquely associated with spectral pathology in the LIFC (TFCE; *p*_FWE_ = 0.027; peak vertex = *x*: −51, *y*: 36, *z*: 2; Fig. 3A), with greater LIFC deviations associated with greater speech impairment (Fig. 3B). This effect was normalized in individuals taking higher daily equivalent doses of dopamine replacement therapy (*N* = 25; *t*(18) = 2.43, *p* = 0.026; Supplementary Fig. 4). Neural activity in all tested frequency bands (from delta to beta) contributed to this effect (ΔAIC; delta = 10.56; theta = 2.86; alpha = 9.74; beta = 3.80; Fig. 3C), with the strongest influences from the delta and alpha bands. Post-hoc analysis of frequency-specific relationships suggested that speech impairment was related to an acceleration of neural activity in the LIFC: greater impairment was associated with increased activity in the faster (alpha & beta) and decreased activity in the slower (delta & theta) frequency bands (Fig. 3D and Supplementary Fig. 5). SDI values in the LIFC did not significantly relate to clinical motor function (UPDRS-III, minus speech sub-scores; *t*(51) = 1.54, *p* = 0.130) or MoCA (*t*(56) = −0.25, *p* = 0.807) scores. The relationship between cross-spectral pathology and articulation impairment remained significant (*p* < 0.001) when potential confounds were added to the model (head motion: *p* = 0.284; eye movements: *p* = 0.147; cardiac artifacts: *p* = 0.996; local cortical thickness: *p* = 0.211; local aperiodic slope: *p* = 0.914; disease duration: *p* = 0.282; distance from MEG sensor array: *p* = 0.067), when only French speakers were considered (*N* = 51; *p* < 0.001), and when the SDI metric was computed from all spectral power density estimates in the 2–30 Hz range (*p* = 0.002; Supplementary Fig. 2B). There was no moderating effect of dopamine agonist use on this relationship (*p* = 0.746).

### LIFC-somatomotor beta-frequency connectivity mediates cognitive contributions to articulation impairments in PD

**Fig. 4:**
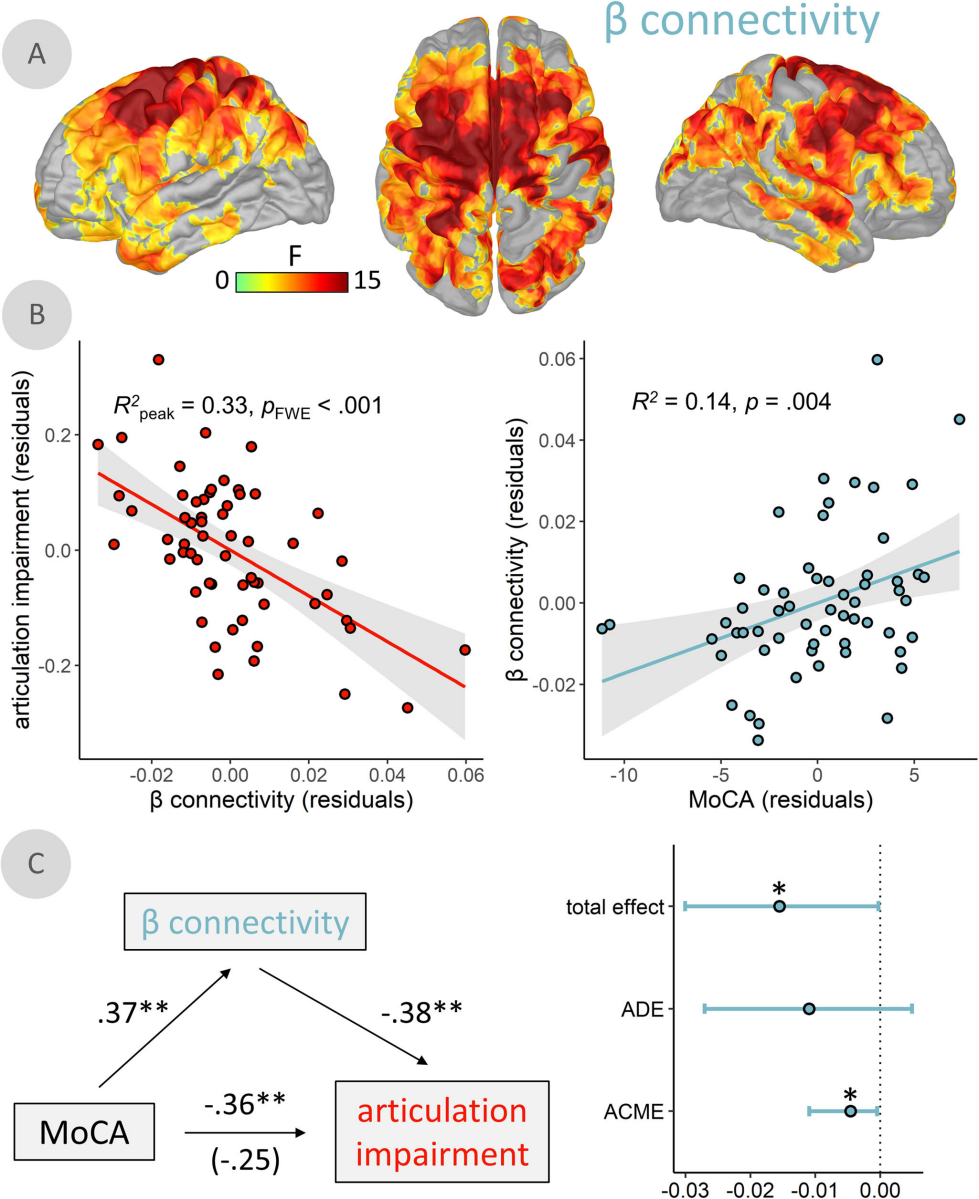
β-frequency functional connectivity between left inferior frontal cortex and somatomotor regions mediates the impact of cognitive decline on articulation impairments. **A** Surface maps indicate a significant relationship between articulation impairment ratings and frequency-resolved functional connectivity, computed using orthogonalized amplitude envelope correlations (AEC) with the peak vertex of the relationship from Fig. 3 as the seed. **B** β-connectivity and articulation impairments from the peak vertex of this relationship are plotted on the left, and the relationship between the same β-connectivity values and cognitive function (i.e., Montreal Cognitive Assessment [MoCA] scores) are plotted on the right. **C** Paths between MoCA scores, articulation impairment ratings, and β-connectivity values to the left indicate partial correlations (*r*) between each set of variables. The correlation value in parentheses represents the mediated relationship between MoCA scores and articulation impairment ratings when accounting for β-connectivity. The total effect, average direct effect (ADE), and average causal mediation effect (ACME; i.e., indirect effect) are plotted with 95% confidence intervals to the right. All models controlled for age. Shaded intervals represent the 95% confidence interval. **p < 0.01, *p < 0.05.

We computed whole-cortex frequency-specific functional connectivity maps to examine whether signal similarities between the LIFC and the rest of the cortex were related to speech impairments in PD. The connectivity analyses were seeded at the vertex location corresponding to the peak of the SDI-articulation impairment statistical map (Fig. 3A; x: −51, y: 36, z: 2). We found that articulation impairment related to connectivity between LIFC and a distributed network of pre-motor, anterior cingulate, and somatomotor regions in the beta band, above and beyond the effects of age, prosody impairments, and voice impairments (TFCE across vertices and secondary Bonferroni correction across 4 frequencies; *p*_FWE_ < 0.001; peak vertex = x: −11, y: −12, z: 46; Fig. 4A). This relationship was such that patients with weaker LIFC-somatomotor functional connectivity exhibited worse articulation impairments (Fig. 4B, left). This relationship remained significant (*p* < 0.001) when potential confounds were added to the model (head motion: *p* = 0.749; eye movements: *p* = 0.517; cardiac artifacts: *p* = 0.970; disease duration: *p* = 0.502) and when only French speakers were considered (*N* = 51; *p* < 0.001). There were no moderating effects of dopamine agonist use (*p* = 0.501) or levodopa equivalent daily dose (*p* = 0.843) on this relationship. Further, this shared variance was independent of SDI effects in the LIFC, as both LIFC SDI values (*t*(56) = 4.28, *p* < 0.001) and beta LIFC-somatomotor connectivity (*t*(56) = −5.66, *p* < 0.001) were significantly associated with articulation impairment, above and beyond the other, when included in a single linear model (*R*^*2*^ = 0.52). Beta LIFC-somatomotor functional connectivity did not relate to UPDRS III scores (*t*(51) = −1.10, *p* = 0.275), but did significantly covary with MoCA scores (*t*(56) = 2.98, *p* = 0.004; Fig. 4B, right), such that stronger connectivity was related to better cognitive function. The relationship between MoCA scores and articulation impairment was also fully mediated by beta-band connectivity (causal mediation analysis, 10,000 simulations; Fig. 4C), as indicated by a significant indirect effect (average causal mediation effect = −0.005, *p* = 0.024) and a non-significant direct effect (average direct effect = −0.011, *p* = 0.223) upon addition of connectivity values to the linear model.

## Discussion

This study introduces and combines novel approaches to spectral brain mapping and speech impairment quantification to delineate the functional neural pathology contributing to speech impairment in PD. In one of the largest MEG studies of PD to date, we identify a pathological relationship between articulation impairments and spectral deviations in the LIFC, with strongest contributions from neurophysiological activity in the delta and alpha bands. In healthy adults, the LIFC is a hub that exhibits multi-frequency interactions with a number of language network regions^45^. Our data also showed that neurophysiological connectivity between LIFC and a network of somatomotor cortices in the beta band were independently associated with articulation impairments, and fully mediated the effect of cognitive abilities on these impairments. Together, these results provide a spatially- and spectrally-resolved cortical network that explained 52% of the variance in articulatory impairments in our sample of patients with PD. These findings may be of significance to future biomarker research and therapeutic targeting in PD, although further research into the out-of-sample predictive capacity of the SDI is required before this approach could be applied in the clinic. We also anticipate that our new, individualized modeling approach of spectral brain pathology for each patient may translate and be meaningful to other clinical populations.

Our approach to speech impairment quantification is predicated on the notion that clinical endpoints for speech production studies in PD need to improve speech perception by human raters^17–19,75,76^. One novel contribution of the present study is the rating of speech features by non-experts through an intuitive and interactive app (*audio_tokens*)^74^, that facilitates simultaneous comparison of speech samples from multiple study participants. Despite being obtained from non-experts, the ratings exhibited a degree of inter-rater reliability comparable to those from gold-standard procedures in the field, such as speech intelligibility quantification using transcription^76^. The *audio_tokens* ratings related to both non-speech motor features (i.e., UPDRS-III with speech sub-scores subtracted) and cognitive function (i.e., MoCA scores) more effectively than common acoustical features derived from automated methods. The *audio_tokens* ratings obtained remotely from non-experts were also similar to the assessments performed in the clinic by a trained professional (i.e., the speech sub-score of the UPDRS-III). We believe these findings establish the validity and potential utility of the *audio_tokens* approach in patients with PD, particularly since comparable speech samples can be easily collected and rated without in-person visits to the clinic. Pending further out-of-sample validation of this approach, the significance is that of tele-medical, longitudinal patient evaluations. A thorough comparison to more advanced automated speech quality quantification procedures (e.g., based on machine-learning techniques)^77^ remains warranted, albeit beyond the scope of this study. These comparisons should also be extended with future studies into the out-of-sample predictive capacity of each speech quantification approach. If these comparisons favor our approach, the additional predictive clinical power of human speech ratings can be harnessed to improve automated speech extraction methods, which in turn can alleviate the costs of and limited access to movement disorder specialists.

Using a novel synoptic analytical approach to map multi-frequency neurophysiological effects, we found a topographic pattern that aligns with previous research in patients with PD^67^, wherein the strongest and most consistent disease-related spectral deviations are expressed along the somato-motor cortices. Prefrontal regions showed the highest inter-patient variability. We validate this new approach in two ways. First, we show that the SDI is a better model of clinically-relevant neurophysiological changes in PD than a traditional band-limited approach in virtually every region of the brain. We interpret this finding as indicative of the fact that the neurophysiological deviations that have been related to motor and cognitive dysfunction in previous studies of PD (e.g., reduced beta oscillations in relation to motor impairments) are captured by the synoptic SDI metric, alongside additional information that is shared across frequency bands. We also show that the SDI approach is not sensitive to the definition of canonical frequency-band limits nor to the frequency resolution of the power spectrum. Taken together, these data provide evidence for the reliability, stability and practical utility of the SDI metric, with anticipated further improvements, including the use of varying similarity/distance metrics in the SDI computation.

By relating speech impairments to spectral deviations from healthy neurophysiological activity, we identified that the greater the spectral deviations in LIFC, the more pronounced the articulation deficits in patients with PD. The LIFC is a key node of the speech production network^21–23,78^ associated to the metrical encoding of to-be-produced speech representations^78,79^. Previous research^80,81^ also suggests that the anterior location of this effect along the inferior frontal gyrus might indicate a deficit in the semantic and/or lexical aspects of articulatory functions in PD. Further, the slower range of neurophysiological activity involved (delta & theta frequency bands) is associated to temporal expectation and parsing mechanisms of sensory inputs^42,82–85^. Previous research has shown that discrimination of rhythmic auditory stimuli is impaired in patients with Parkinson’s disease^86^. Patients with PD also exploit metrical regularities more than healthy participants when entraining their speech to another person’s^87^. As such, temporal processing deficits have been proposed as a target for ameliorating the language deficits often seen in patients with PD^88,89^. We thus interpret the present observations as patients experiencing difficulties producing clear and precise speech sounds because of impaired mechanisms for parsing and rhythmically encoding phonological information prior to speech motor initiation. We also found that increased expressions of faster frequencies (alpha & beta frequency bands) in LIFC were related to speech deficits. Strong alpha/beta activity in the parieto-occipital^90,91^ and somatomotor^92–94^ cortices reflects enhanced functional local inhibition. Additionally, alpha/beta activity in LIFC is reduced around speech preparation^50,95,96^. Taken together, we interpret our findings as indicative that patients with reduced inhibition of LIFC at rest exhibited the best articulation abilities, possibly because of greater ability to parse speech information in time. Overall, these effects involve at once several components of the neurophysiological frequency spectrum in a single brain region. This highlights the utility of the proposed spectral deviation approach, as this pattern of shared speech impairment information across multiple frequencies would not have been detected using a band-limited analysis (e.g., focusing on the beta band).

In contrast to the multi-spectral and spatially-focal nature of speech-related neural pathology in the LIFC, the network-level connectivity patterns that related to articulation abilities in patients with PD were limited to the beta-band and spatially-widespread. Our data show that beta-band functional connectivity between the LIFC and prefrontal, frontal, and parietal somatomotor regions was negatively associated with speech impairments in patients. Substantial structural and functional connectivity effects have been reported between these regions^97,98^, and functional connectivity between LIFC and superior frontal regions during speech comprehension is reduced in PD^99,100^. Previous studies have also reported increased beta-band connectivity in patients with PD^67^, with indications of clinical significance, although it is still unclear if the relationship is one of impairment^101,102^ or compensation^72,103–105^. Our results point at a possible compensation role for beta-band connectivity in speech production: in the tested patient cohort, the stronger the LIFC-somatomotor beta connectivity, the higher the cognitive and articulation abilities. This effect was statistically independent of the spectral deviations we observed in LIFC, which indicates that both effects represent distinct PD functional pathologies of speech production brain systems. We also note that increases in beta-frequency power and connectivity do relate to clinical impairments, albeit in opposing directions: increased power and decreased fronto-motor connectivity are both associated with worse speech impairments. Differing patterns of PD-related change in beta-frequency amplitude versus connectivity have been shown previously^67,73,101,102^. Our findings indicate that these opposing neurophysiological effects of the disease are also inversely related to clinical outcomes. We hope that these findings can inspire future research of individualized clinical monitoring and interventions via, for example, non-invasive therapeutic neuromodulation.

Our data also show that the strength of beta-band connectivity effects fully mediates the relationship between cognitive abilities (MoCA scores) and articulation impairments. In other words, the contribution of cognitive declines to speech impairments in Parkinson’s disease seems to be entirely (but not necessarily solely) accounted for by variations in LIFC-somatomotor connectivity in the beta band. However, we did not observe such an effect between motor impairments and speech deficits, nor any significant relationship between UPDRS-III scores and beta-band connectivity. Our interpretation is that reduced beta-band connectivity between LIFC and somatomotor cortices is a biological proxy for cognitive contributions to articulation deficits in PD.

Whether speech impairments are “dopa-resistant”^106^ remains controversial^107,108^. We find a normalization of the pathological relationship between spectral deviations and articulatory impairments in the LIFC of patients with higher equivalent dopamine therapy doses, which indicates that the neurophysiological bases of speech deficits in PD may be, in part, modifiable with levodopa. The nature of this effect also suggests that the spectral deviations and related speech impairments that we observe are not a secondary result of dopamine replacement therapy. However, we acknowledge that the present data cannot fully disentangle which of these effects are related to PD neuropathology versus the use of dopamine replacement therapies. All patients in our study were under a stable dosage of antiparkinsonian medication, but only a small subset (*N* = 25) had detailed data available regarding their medication regimens. We foresee the present data will inspire further research to clarify these aspects. As the vast majority of individuals diagnosed with PD are administered antiparkinsonian drugs, we argue that reporting both medication-related and -unrelated effects advance research against the disease.

The relatively large and heterogeneous patient data used for the present study were aggregated from the Quebec Parkinson Network (QPN). The open repository features minor inconsistencies in the availability of clinical information across participants. Specifically, non-overlapping subsets of participants who underwent MEG neuroimaging did not have details available regarding dopaminergic medication regimens, Hoehn & Yahr staging, Unified Parkinson Disease Rating Scale (UPDRS) subscale scores, time since diagnosis, or whether they were taking dopamine agonists. Although we acknowledge this is a limitation of the current study, we feel that the scientific benefits of this unique dataset do stand out. We also emphasize that we did assess the impact of these clinical features by testing their relationships to our key findings, with participants with missing data excluded pairwise.

In sum, we believe our data advance the understanding of basic mechanisms involved in speech production in health and disease. They also highlight two new dissociable neurophysiological markers of symptom-specific clinical decline in PD, which should be validated in future studies of their out-of-sample predictive capacity. If validated, these biomarkers could then be used to improve the targeting of non-invasive neuromodulatory therapies in PD^69,71^. Further, the principle of the SDI is generalizable to other neurological and/or psychiatric disorders. In particular, the combination of easily administered speech sample recordings with a short resting-state MEG session has potential for identifying biomarkers in a host of neurodegenerative and neurodevelopmental disorders, many of which have speech impairment as a presenting complaint.

## Methods

### Participants

The Research Ethics Board at the Montreal Neurological Institute reviewed and approved this study. Written informed consent was obtained from every participant following detailed description of the study, and all research protocols complied with the Declaration of Helsinki. Exclusionary criteria for all participants included current neurological (other than PD) or psychiatric disorder; MEG contraindications; and unusable MEG, speech sample, or demographic data. All participants completed the same speech and MEG protocols with the same instruments at the same site.

Patients with mild-to-moderate idiopathic PD were enrolled as a part of the Quebec Parkinson Network (QPN; https://rpq-qpn.ca/)^109^ initiative, which includes extensive clinical, neuroimaging, neuropsychological, and biological profiling of participants. A final sample of 59 participants with PD fulfilled the criteria of having complete and useable MEG, speech sample, and demographic data. All patients with PD were prescribed a stable dosage of antiparkinsonian medication with satisfactory clinical response prior to study enrollment. Twenty-five of these patients opted to provide detailed medication regimen information, from which the Levodopa Equivalent Daily Dose was calculated using established conversion factors^110^ and used in *post hoc* analyses. Patients were instructed to take their medication as prescribed before research visits, and thus all data were collected in the practically-defined “ON” state. The motor subtest of the Unified Parkinson’s Disease Rating Scale (UPDRS-III)^111^ and Montreal Cognitive Assessment (MoCA)^112^ were administered to all participants by a trained clinician administrator (S.D.). We also computed all relationships between motor pathology and speech feature ratings with the clinical speech sub-score (UPDRS-III, item 1; sub-scores available for *N* = 54) subtracted from the UPDRS-III scores to avoid bias. Additional clinical data were also available for subsamples of the patient group in the form of the Hoehn & Yahr scale (N = 48) and disease duration (i.e., time since diagnosis; *N* = 56).

Neuroimaging data from 65 healthy older adults were collated from the QPN (*N* = 10), PREVENT-AD (*N* = 40)^113^, and OMEGA (*N* = 15)^114^ data repositories to serve as a comparison group for the patients with PD. These participants were selected so that their demographic characteristics, including age (Mann–Whitney *U* test; *W* = 1551.50, *p* = 0.067), self-reported sex (chi-squared test; χ^2^ = 0.61, *p* = 0.434), handedness (chi-squared test; χ^2^ = 0.29, *p* = 0.863), and highest level of education (Mann–Whitney *U* test; *W* = 1831.50, *p* = 0.615), did not significantly differ from those of the patient group. Importantly, due to the marginal difference observed between groups, age was included as a nuisance covariate in all statistical models. This sample size was selected based on previous work indicating that group sizes of *N* > 50 are sufficient to estimate frequency-specific neurophysiological activity in healthy adults^115^. Group demographic summary statistics and comparisons, as well as clinical summary statistics for the patient group, can be found in Table 1.

### Speech sample collection, rating & processing

Four auditory speech samples of cued sentence repetitions were recorded from each patient by the same neuropsychologist. Participants were fluent in French and/or English, and were allowed to hear and speak the sentences for repetition in the language of their choice (French: *N* = 51; English: *N* = 8). Speech samples were recorded an average of 19.63 days (SD = 53.44) from the date of neuroimaging data collection. All patients repeated four sentences (two easy, two hard; to capture a range of difficulties and avoid potential ceiling effects) in the same order (see Supplementary Information: Materials & Methods). Sentences were taken from an in-house neuropsychological battery at the Montreal Neurological Institute, and were matched between languages in terms of length and number of syllables. To ensure that the language spoken did not meaningfully bias our analyses, we reproduced our major findings excluding the English speakers (*N* = 8) from our sample. Sentences were pre-recorded by a female native speaker of each language and played to the participants at test time. Speech samples were recorded from participants using a Shure SM10ACN Cardioid Dynamic head-worn microphone. The microphone was positioned such that it was comfortable for the participants to wear, approximately at a two-finger distance to the participant’s mouth. Recordings were performed using a Tascam DR-10L Digital Audio Recorder.

The speech features of the recordings were then quantified in two ways: (1) using an automated extraction approach based on commonly-used metrics from previous literature in PD^4,116,117^, and (2) using the new Javascript toolbox *audio_tokens*^74^ to collect speech impairment ratings from non-experts. The automated extraction approach quantified the following features of the speech data using Praat^118^ with the *Python-parselmouth* interface^119^: harmonics-to-noise ratio (hnr), timing and amplitude fluctuations of glottal pulses (jitter and shimmer, respectively), standard deviation of pitch measured over voiced segments (f0_std), and a proxy for vowel space (area), namely the product of the inter-quartile range of F1 and F2 values measured over voiced segments (as a simplified version of the procedure described in Sandoval et al.^116^). To derive a single measure roughly representing voice quality from these features, the first principal component was extracted from the hnr, jitter, and shimmer features. The resulting metric, f0_std, and area were used in further analyses.

For the non-expert ratings of the speech samples, three university students with minimal-to-no experience in speech assessment each rated multiple features of every sample on a continuous scale, including the magnitude of impairments in voice (instruction: “*Does the speaker’s voice sound harsh or breathy?*”), articulation (instruction: “*Is the speaker’s articulation slurred or imprecise?*”), and prosody (instruction: “*Does the speech sound flat or monotonous?*”). Sample ratings were performed in the *audio_tokens* toolbox^74^ (Fig. 1A), which allowed for interactive and dynamic comparison of speech samples across patients. The resulting values were then averaged across the 4 sentences for each speech feature/rater/patient, and the intraclass correlation coefficient (ICC; type [C,k]: multiple raters, two-way random effects, consistency)^120^ was computed to assess inter-rater reliability for each feature. Given the high consistency across raters for all three features (Fig. 1A), we used the mean of these values across the 3 raters to derive singular estimates of speech impairment for each feature in every patient. The speech sub-score of the UPDRS-III was also used as a measure of speech impairment collected by a trained clinician administrator.

### Magnetoencephalography data collection, preprocessing & analysis

Eyes-open resting-state MEG data were collected from each participant using a 275-channel whole-head CTF system (Port Coquitlam, British Columbia, Canada) at a sampling rate of 2400 Hz and with an antialiasing filter with a 600 Hz cut-off. Noise-cancellation was applied using CTF’s software-based built-in third-order spatial gradient noise filters. Recordings lasted a minimum of 5 min^115^ and were conducted with participants in the seated position as they fixated on a centrally-presented crosshair. Participants were monitored during data acquisition via real-time audio-video feeds from inside the shielded room, and continuous head position was recorded for each session.

MEG preprocessing was performed in *Brainstorm*^121^ unless otherwise specified, with default parameters and following good-practice guidelines^122^. The data were bandpass filtered between 1–200 Hz to reduce slow-wave drift and high-frequency noise, and notch filters were applied at the line-in frequency and harmonics (i.e., 60, 120 & 180 Hz). Signal space projectors (SSPs) were derived around cardiac and eye-blink events detected from ECG and EOG channels using the automated procedure available in *Brainstorm*^123^, reviewed and manually-corrected where necessary, and applied to the data. Additional SSPs were also used to attenuate highly-stereotyped artifacts on an individual basis. Artifact-reduced MEG data were then arbitrarily epoched into non-overlapping 6 second blocks and downsampled to 600 Hz. Data segments still containing major artifacts (e.g., SQUID jumps) were excluded within each session using the union of two standardized thresholds of ±3 median absolute deviations from the median: one for signal amplitude and one for gradient. An average of 79.19 (SD = 14.68) epochs were used for further analysis (patients: 84.07 [SD = 7.78]; controls: 74.77 [SD = 17.82]). Empty-room recordings lasting approximately 2 min were collected on or near the same day as the data recordings and were processed using the same pipeline, with the exception of the artifact SSPs, to model environmental noise statistics for source analysis.

MEG data were coregistered to each individual’s segmented T1-weighted MRI (Freesurfer *recon-all*)^124^ using approximately 100 digitized head points. For participants without useable MRI data (*N* = 11 patients with PD; *N* = 3 healthy adults), a quasi-individualized anatomy was created and coregistered to the MEG data by warping the default Freesurfer anatomy to the head digitization points and anatomical landmarks for that participant^125^. Source imaging was performed per epoch using individually-fitted overlapping-spheres forward models (15,000 vertices, with current flows unconstrained to the cortical surface’s normal direction) and dynamic statistical parametric mapping (dSPM). Noise covariance estimated from the previously-mentioned empty-room recordings were included in the computation of the dSPM maps.

Inspired by previously-developed measures of multi-spectral neurophysiological signal pathology^126,127^ and cortical morphometric similarity in clinical populations^128,129^, we developed a new metric of neurophysiological spectral pathology derived from time-resolved MEG source maps: the Spectral Deviation Index (SDI; Fig. 2A). The SDI provides an estimate of deviations from healthy levels of neurophysiological activity across multiple frequency bands, simultaneously. This estimate is computed per patient and retains the original spatial resolution of the MEG cortical maps, thus allowing for unbiased detection of functional neurophysiological pathology without strong a priori hypotheses regarding the frequency bands involved. This approach preserves statistical sensitivity and does not require corrections for multiple comparisons across multiple frequency bands. It also enables the detection of multi-spectral patterns of neurophysiological changes. We computed vertex-wise estimates of power spectral density from the source-imaged MEG data using Welch’s method (3 s time window, 50% overlap), which we then averaged over canonical frequency bands (delta: 2–4 Hz; theta: 5–7 Hz; alpha: 8–12 Hz; beta: 15–29 Hz)^123^, and over all artifact-free 6-second epochs for each participant. The root-mean-square (RMS) norm of PSD across the three unconstrained orientations at each vertex location and for each participant was then projected onto a template cortical surface (*FSAverage*) for comparison across participants. For each PD participant, the resulting PSD map of spectrally-resolved estimates of neural power was correlated across frequencies (i.e., delta, theta, alpha and beta) at every spatial location (i.e., vertex) with the comparable estimates from each control participant. This resulted in a matrix of Pearson correlation coefficients (*r*) representing the spectral neurophysiological similarity between all patient and control participants at each cortical location. Using these matrices, we generated for each patient the continuous SDI metric of spectral deviations per vertex by taking the median of the resulting Pearson coefficients (*r*) across correlations with all control participants, normalizing these values using the Fisher transform (i.e., the inverse hyperbolic tangent; using the *atanh* function in Matlab), and subtracting them from 1 to generate a normally-distributed metric of spectral deviation (i.e., higher values indicate greater functional neural pathology). A similar approach was taken using all spectral power estimates in the 2–30 Hz frequency range (i.e., without averaging over canonical frequency bands; 28-Hz range * 1/3 Hz frequency resolution = 85 samples) to ensure the stability of the SDI computation against differences in frequency-band limits and sparse spectral sampling.

In addition to deriving individual SDI maps, we also used the source-imaged MEG data to investigate patterns of connectivity that relate to speech impairments in patients with PD. We extracted the first principal component from the three elementary source time series at each vertex location in each participant’s native space, and derived whole-cortex functional connectivity maps, using the peak vertex identified in our spatially-resolved SDI statistical analysis (back-transformed into each participant’s native space) as the seed. We used orthogonalized amplitude envelope correlations (AEC)^130,131^ as the connectivity measure, based on the same frequency definitions used for the SDI mapping. We estimated connectivity over each epoch and averaged the resulting AEC estimates across epochs, yielding a single AEC map per participant and frequency band. We projected these individual AEC maps onto the same template cortical surface (*FSAverage*) for group analyses.

Finally, we extracted several metrics to test for potential confounds of our primary effects of interest. To test whether our findings were mediated by local neurodegeneration, we estimated cortical thickness with the *recon-all* pipeline in *Freesurfer*^124^ and extracted values at the peak vertex of each significant statistical cluster for inclusion as nuisance covariates in post-hoc models. To determine whether SDI effects were related to shifts of the aperiodic broadband component of neural spectra, we processed PSDs with *specparam* (*Brainstorm* MATLAB version; frequency range = 2–40 Hz; Gaussian peak model; peak width limits = 0.5–12 Hz; maximum n peaks = 3; minimum peak height = 3 dB; proximity threshold = 2 standard deviations of the largest peak; fixed aperiodic; no guess weight)^132^ to estimate the slope of aperiodic neural spectral components. We also investigated possible confound effects due to participant head motion, eye movements, and heart-rate variability: we extracted the RSS of signals from the head position indicators, EOG, and ECG channels, respectively. To measure the impact of the distance of participant’s heads from the sensor array on the SDI outcome, we computed the average Euclidean distance from each participant’s (back-transformed) LIFC peak vertex location to all sensors in the MEG array. Alongside age and disease duration, these derivations were included in *post hoc* statistical models to examine the robustness of the initial effect(s) of interest against potential confounds.

### Statistical analyses

We assessed relationships between continuous variables using the *lm* function in *R*^133^, with a significance threshold of *p* < 0.05. Model comparisons were performed using the Akaike information criterion (AIC), with differences in AIC (ΔAIC) between tested models of | ΔAIC | > 2 considered as meaningful^134^. Where appropriate^135^, we performed causal mediation analyses using a non-parametric bootstrapping approach for indirect effects with 10,000 simulations^136^. This approach can be used to determine to what degree a third factor (M) is responsible for the effect of an independent variable (X) on a dependent variable (Y), by dissecting the original relationship into its direct (X→Y) and indirect (X→M→Y) effects. All statistical models included age as a nuisance covariate. Participants with missing data were excluded pairwise per model.

We performed statistical comparisons using spatially-resolved neural data, covarying out the effect of age, using *SPM12*. Initial tests used parametric general linear models to investigate relationships with speech impairment ratings (i.e., multiple regression with voice, prosody, and articulation impairment ratings as predictors), beyond the effects of age. Contrasts for each speech feature were thus corrected for age and independent of the other features. We used Threshold-Free Cluster Enhancement (TFCE; E = 1.0, H = 2.0; 5000 permutations)^137^ to correct the resulting *F*-contrasts for multiple comparisons across vertices. TFCE avoids the assumptions of parametric modeling, accounts for potential non-uniform spatial autocorrelation of the data, and avoids the arbitrary selection of cluster-defining thresholds. We applied a final cluster-wise threshold of *p*_FWE_ < 0.05 to determine statistical significance, and used the TFCE clusters at this threshold to mask the original statistical values (i.e., vertex-wise *F* values) for visualization. A secondary Bonferroni correction was applied across cluster *p*-values when multiple models were computed with overlapping hypotheses (i.e., in the case of the four frequency-defined connectivity models). We extracted data from the vertex exhibiting the strongest statistical relationship in each cluster (i.e., the “peak vertex”) for subsequent analysis and visualization.

To determine the relative contribution of individual frequency bands to the significant SDI-speech relationships, we used an adapted leave-one-out approach. We recomputed SDIs at each peak-vertex four times – each time excluding data from one frequency band. Speech impairment ratings were then regressed on these modified SDI values, and we derived the ΔAIC between each leave-one-out model and the original model, with higher values indicating greater contribution to the original effect, and a standard cut-off of | ΔAIC | > 2 was used to indicate meaningful contribution. Lines-of-best-fit were also fitted to the data *post hoc* and plotted to display the nature of the underlying relationships between spectral power and speech ratings for each frequency band.

We compared the SDI metric to a canonical band-limited approach of modeling clinical variability. To perform such a comparison across the entire cortical surface, we parcellated the SDI and band-limited source maps (per participant) into 68 regions-of-interest using the Desikan-Killiany atlas^138^ and tested four models per region: two models based on motor scores (i.e., UPDRS-III) and two based on cognitive scores (i.e., MoCA) as dependent variables. For each of these dependent variables, we tested one model using SDI values and another model based on band-limited spectral power as independent variables. For every pair of SDI and band-limited models, ΔAIC was computed by subtracting the AIC of the band-limited model (form: *clinical scores* ~ *delta* + *theta* + *alpha* + *beta* + *age*) from the AIC of the SDI model (form: *clinical scores* ~ *SDI* + age), resulting in a value where lower ΔAIC indicates stronger evidence for the SDI model. The resulting ΔAIC values were thresholded using a standard cut-off of | ΔAIC | > 2 and plotted for visualization using *ggseg*^139^.

### Reporting summary

Further information on research design is available in the Nature Research Reporting Summary linked to this article.

## Supplementary information

Supplementary Information

Reporting Summary

## Data Availability

Data used in the preparation of this work are available from the QPN through the Clinical Biospecimen Imaging and Genetic (C-BIG) repository (https://www.mcgill.ca/neuro/open-science/c-big-repository)^109^, the PREVENT-AD open resource (https://openpreventad.loris.ca/)^113^, and the OMEGA repository (https://www.mcgill.ca/bic/resources/omega)^114^. Code for MEG preprocessing and the spectral deviation analysis is available at https://github.com/aiwiesman/QPN_SpeechAnalysis. Rejection of epochs containing artifacts was performed with the *ArtifactScanTool* (https://github.com/nichrishayes/ArtifactScanTool).
