# Supplementary material for: Aberrant neurophysiological signaling associated with speech impairments in Parkinson’s disease
Source: NPJ Parkinsons Dis. 2023 Apr 14;9:61. doi: 10.1038/s41531-023-00495-z (PMC10104849; doi:10.1038/s41531-023-00495-z)
Supplement: Supplementary file 1 — Supplementary Information [file 41531_2023_495_MOESM1_ESM.pdf]

Supplementary Information for *Aberrant neurophysiological signaling associated with speech impairments in Parkinson's disease*

Materials & Methods

Sentences for Repetition

English:

- *The mother washes her child*. (easy)
- *Her hair got tangled in the wind.* (easy)
- *I can't remember where I put my brand new bag* (hard)
- *They caught some trout while they were fishing on the lake* (hard)

French:

- *Je cherche un nouveau manteau.* (easy)
- *Je veux le plus petit morceau.* (easy)
- *Les chevaux ont eu très peur lors de l'incendie.* (hard)
- *Le gros chien a attaqué l'homme très soudainement.* (hard)

Supplementary Figures

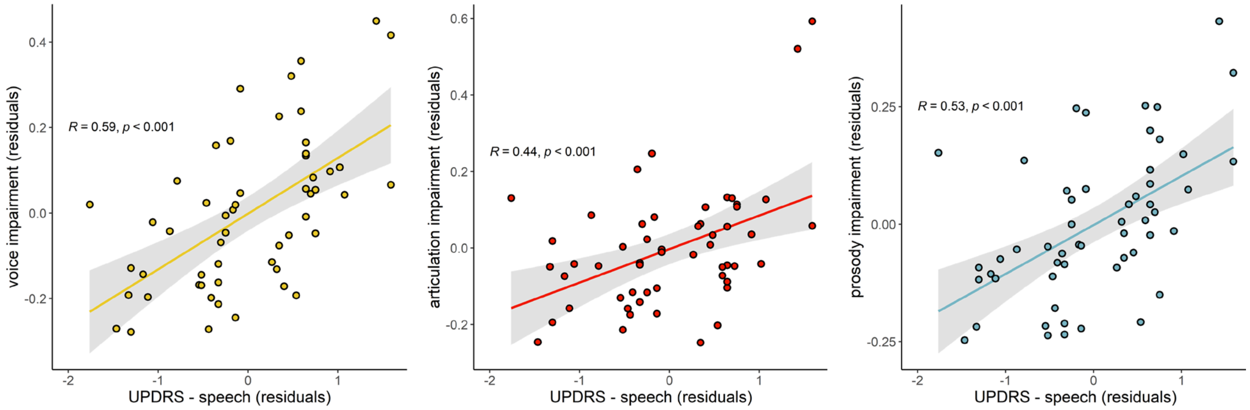

Supplementary Figure 1. **Non-expert speech impairment ratings are associated with clinical motor speech assessments.** Significant linear relationships between voice, articulation, and prosody impairments and the speech sub-score of the Unified Parkinson's Disease Rating Scale part III. All models controlled for age. Shaded intervals represent the 95% confidence interval.

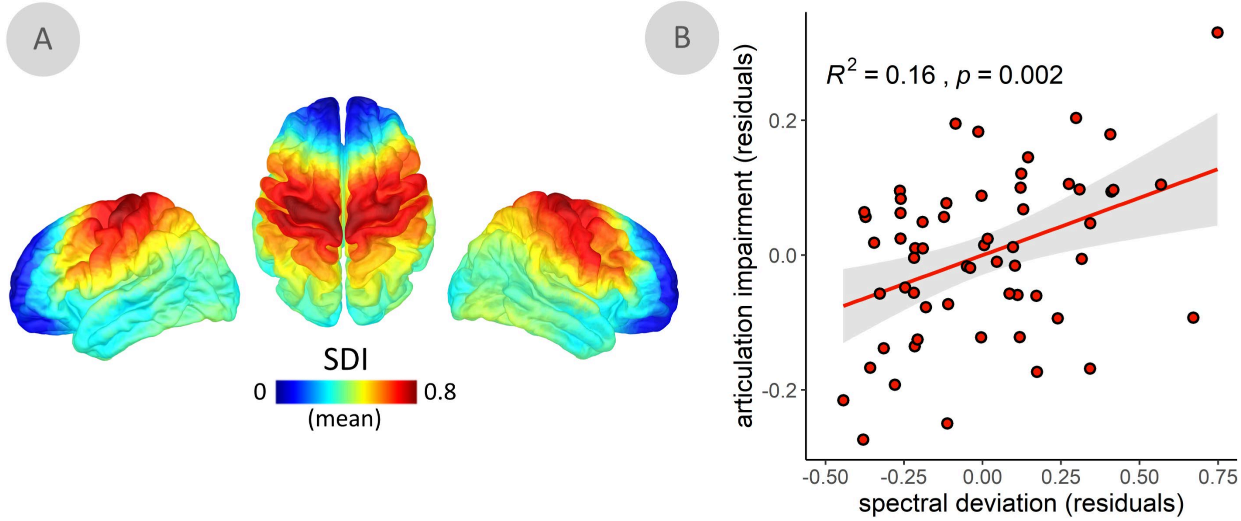

Supplementary Figure 2. Spectral deviations computed using all spectral power densities from 2 – 30 Hz. Spectral Deviation Index (SDI) values computed using all spectral power density estimates from 2 – 30 Hz exhibit similar (A) spatial patterns and (B) relationships to articulatory impairments as those seen in Figures 2B and 3B, respectively.

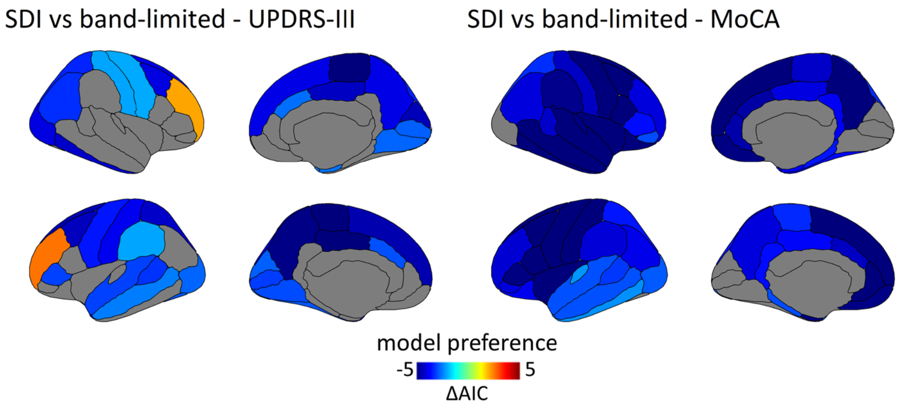

Supplementary Figure 3. The Spectral Deviation Index (SDI) outperforms a band-limited model of cognitive and motor impairments. Per each region of the Desikan-Killiany atlas, model comparisons were performed between the SDI model and a band-limited model (i.e., with spectral power in all four frequencies as independent variables), with clinical function as the dependent variables (UPDRS-III, left; MoCA, right), covarying the effect of age. Differences in the Akaike Information Criterion from these model comparisons are plotted per each region, with a standard threshold of $|\Delta AIC| > 2$ applied. In virtually every region of the brain, the SDI model outperformed the band-limited model, as indicated by $\Delta AIC < -2$.

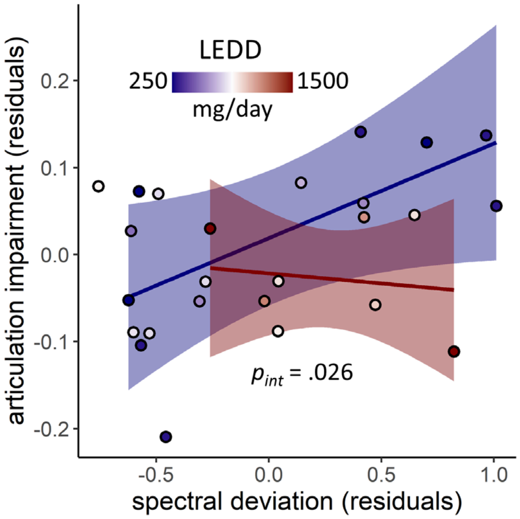

Supplementary Figure 4. Levodopa Equivalent Daily Dose (LEDD) moderates the relationship between left inferior frontal spectral deviations and articulatory impairments. In a subset of patients with Parkinson's disease for whom detailed medication regimen information was available (N = 25), LEDD was found to significantly moderate the relationship between the Spectral Deviation Index (SDI) and articulatory impairments shown originally in Figure 3B. This interaction effect was such that patients taking a larger equivalent dose of levodopa exhibited a weaker positive relationship, potentially indicating a normalizing effect of dopamine replacement therapy. LEDD was treated as a continuous variable in the linear model, however, for visualization purposes lines-of-best fit and 95% confidence intervals are plotted for the first and third tertiles.

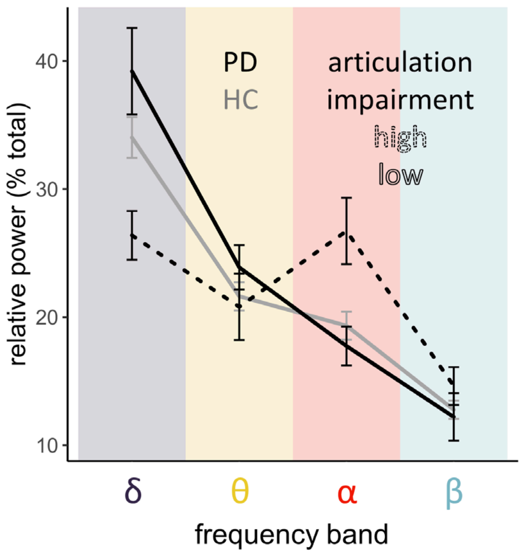

Supplementary Figure 5. Frequency-wise differences in left inferior frontal cortex (LIFC) spectral power as a function of disease status and articulatory impairment severity. Line plots represent the LIFC mean relative spectral power (y-axis) per frequency band (x-axis) for the healthy control participants (grey) and the patients with Parkinson's disease (black). Error bars represent ± 1 standard error of the mean. Patients with Parkinson's disease are further subdivided into high (dotted line, 4th quartile) and low (solid line, 1st quartile) severity of articulation impairments, to highlight the spectral deviations responsible for the relationships shown in Figure 3.

Consortium Authors

A complete listing of **PREVENT-AD Research Group** can be found in the PREVENT-AD database:

https://preventad.loris.ca/acknowledgements/acknowledgements.php?date=[2022-02-01]

The members of the **Quebec Parkinson Network** are listed alphabetically below:

Isabelle Beaulieu-Boire - Université de Sherbrooke, Sherbrooke, Canada
Pierre Blanchet - Université de Montréal, Montréal, Canada
Sarah Bogard - Montreal Neurological Institute, McGill University, Montreal, Canada
Manon Bouchard – Clinique NeuroLévis, Lévis, Canada
Sylvain Chouinard - Université de Montréal, Montréal, Canada
Francesca Cicchetti - CHU de Québec-Université Laval, Québec, Canada
Martin Cloutier - Neuro-Rive-Sud, Longueuil, Canada
Alain Dagher - Montreal Neurological Institute, McGill University, Montreal, Canada
Samir Das - Montreal Neurological Institute, McGill University, Montreal, Canada
Clotilde Degroot - Montreal Neurological Institute, McGill University, Montreal, Canada
Alex Desautels - Université de Montréal, Montréal, Canada
Marie Hélène Dion - Université de Montréal, Montréal, Canada
Janelle Drouin-Ouellet - Université de Montréal, Montréal, Canada
Anne-Marie Dufresne - CHU de Québec-Hôpital de l'Enfant-Jésus, Québec, Canada
Nicolas Dupré - CHU de Québec-Université Laval, Québec, Canada
Antoine Duquette - Université de Montréal, Montréal, Canada
Thomas Durcan - Montreal Neurological Institute, McGill University, Montreal, Canada
Lesley K. Fellows - Montreal Neurological Institute, McGill University, Montreal, Canada
Edward Fon - Montreal Neurological Institute, McGill University, Montreal, Canada
Jean-François Gagnon - Université du Québec à Montréal, Montréal, Canada
Ziv Gan-Or - Montreal Neurological Institute, McGill University, Montreal, Canada
Angela Genge - Montreal Neurological Institute, McGill University, Montreal, Canada
Nicolas Jodoin - Centre hospitalier de l'Université de Montréal, Montréal, Canada
Jason Karamchandani - Montreal Neurological Institute, McGill University, Montreal, Canada
Anne-Louise Lafontaine - Montreal Neurological Institute, McGill University, Montreal, Canada
Mélanie Langlois - CHU de Québec-Université Laval, Québec, Canada
Etienne Leveille - Montreal Neurological Institute, McGill University, Montreal, Canada
Martin Lévesque - Université Laval, Québec, Canada
Calvin Melmed - McGill University, Montreal, Canada
Oury Monchi - Université de Montréal, Montréal, Canada
Jacques Montplaisir - Université de Montréal, Montréal, Canada
Michel Panisset - Université de Montréal, Montréal, Canada
Martin Parent - Université Laval, Québec, Canada
Minh-Thy Pham-An - Hôpital Hôtel-Dieu de Saint-Jérôme, Saint-Jérôme, Canada
Jean-Baptiste Poline - Montreal Neurological Institute, McGill University, Montreal, Canada
Ronald Postuma - Montreal Neurological Institute, McGill University, Montreal, Canada
Emmanuelle Pourcher - CHU de Québec-Université Laval, Québec, Canada
Trisha Rao - McGill University, Montreal, Canada
Jean Rivest - CHU de Québec-Université Laval, Québec, Canada
Guy Rouleau - Montreal Neurological Institute, McGill University, Montreal, Canada

Madeleine Sharp - Montreal Neurological Institute, McGill University, Montreal, Canada
Valérie Soland - CHU de Montréal, Montréal, Canada
Michael Sidel - Montreal Neurological Institute, McGill University, Montreal, Canada
Sonia Lai Wing Sun - Montreal Neurological Institute, McGill University, Montreal, Canada
Alexander Thiel - McGill University, Montreal, Canada
Paolo Vitali - McGill University, Montreal, Canada
