# Supplementary material for: Aberrant neurophysiological signaling associated with speech impairments in Parkinson’s disease
Source: NPJ Parkinsons Dis. 2023 Apr 14;9:61. doi: 10.1038/s41531-023-00495-z (PMC10104849; doi:10.1038/s41531-023-00495-z)
Supplement: Supplementary file 2 — Reporting Summary [file 41531_2023_495_MOESM2_ESM.pdf]

nature portfolio

Corresponding author(s): Sylvain Baillet, PhD
Denise Klein, PhD

Last updated by author(s): Jan 17, 2023

# Reporting Summary

Nature Portfolio wishes to improve the reproducibility of the work that we publish. This form provides structure for consistency and transparency in reporting. For further information on Nature Portfolio policies, see our Editorial Policies and the Editorial Policy Checklist.

## Statistics

For all statistical analyses, confirm that the following items are present in the figure legend, table legend, main text, or Methods section.

| n/a | Confirmed | |
|---|---|---|
| ☐ | ☒ | The exact sample size (*n*) for each experimental group/condition, given as a discrete number and unit of measurement |
| ☐ | ☒ | A statement on whether measurements were taken from distinct samples or whether the same sample was measured repeatedly |
| ☐ | ☒ | The statistical test(s) used AND whether they are one- or two-sided<br>*Only common tests should be described solely by name; describe more complex techniques in the Methods section.* |
| ☐ | ☒ | A description of all covariates tested |
| ☐ | ☒ | A description of any assumptions or corrections, such as tests of normality and adjustment for multiple comparisons |
| ☐ | ☒ | A full description of the statistical parameters including central tendency (e.g. means) or other basic estimates (e.g. regression coefficient) AND variation (e.g. standard deviation) or associated estimates of uncertainty (e.g. confidence intervals) |
| ☐ | ☒ | For null hypothesis testing, the test statistic (e.g. $F$, $t$, $r$) with confidence intervals, effect sizes, degrees of freedom and $P$ value noted<br>*Give P values as exact values whenever suitable.* |
| ☒ | ☐ | For Bayesian analysis, information on the choice of priors and Markov chain Monte Carlo settings |
| ☐ | ☒ | For hierarchical and complex designs, identification of the appropriate level for tests and full reporting of outcomes |
| ☐ | ☒ | Estimates of effect sizes (e.g. Cohen's *d*, Pearson's *r*), indicating how they were calculated |

*Our web collection on statistics for biologists contains articles on many of the points above.*

## Software and code

Policy information about availability of computer code

| | |
|---|---|
| Data collection | Shure SM10ACN Cardioid Dynamic head-worn microphone<br>Tascam DR-10L Digital Audio Recorder<br>CTF 275-channel axial gradiometer MEG system (Port Coquitlam, British Columbia, Canada)<br>Polhemus Fasttrack 3D Digitizer |
| Data analysis | Audio-Tokens Toolbox<br>Python-parselmouth<br>Brainstorm (March 2021 distribution)<br>Custom Matlab code (https://github.com/aiwiesman/QPN_SpeechAnalysis; https://github.com/nichrishayes/ArtifactScanTool)<br>R (version 4.0.3)<br>SPM12 |

For manuscripts utilizing custom algorithms or software that are central to the research but not yet described in published literature, software must be made available to editors and reviewers. We strongly encourage code deposition in a community repository (e.g. GitHub). See the Nature Portfolio guidelines for submitting code & software for further information.

## Data

Policy information about availability of data

All manuscripts must include a data availability statement. This statement should provide the following information, where applicable:

- Accession codes, unique identifiers, or web links for publicly available datasets
- A description of any restrictions on data availability
- For clinical datasets or third party data, please ensure that the statement adheres to our policy

Data used in the preparation of this work are available from the QPN through the Clinical Biospecimen Imaging and Genetic (C-BIG) repository (https://www.mcgill.ca/neuro/open-science/c-big-repository), the PREVENT-AD open resource (https://openpreventad.loris.ca/), and the OMEGA repository (https://www.mcgill.ca/bic/resources/omega).

## Human research participants

Policy information about studies involving human research participants and Sex and Gender in Research.

| | |
|---|---|
| Reporting on sex and gender | Sex was based on self-reporting and was matched across the participant groups. No analyses concerning sex were performed, as this variable was not considered relevant to the experimental hypotheses. |
| Population characteristics | The study sample consisted of 59 patients with mild-to-moderate Parkinson's disease and 65 older adults who were matched on age, self-reported sex, handedness, and highest level of education. All participants were recruited in Canada. |
| Recruitment | Data were aggregated from several open-science initiatives: the Quebec Parkinson Network, the PREVENT-AD open resource, and the OMEGA repository. |
| Ethics oversight | The Research Ethics Board at the Montreal Neurological Institute reviewed and approved this study. |

Note that full information on the approval of the study protocol must also be provided in the manuscript.

# Field-specific reporting

Please select the one below that is the best fit for your research. If you are not sure, read the appropriate sections before making your selection.

☒ Life sciences ☐ Behavioural & social sciences ☐ Ecological, evolutionary & environmental sciences

For a reference copy of the document with all sections, see nature.com/documents/nr-reporting-summary-flat.pdf

# Life sciences study design

All studies must disclose on these points even when the disclosure is negative.

| | |
|---|---|
| Sample size | Parkinson's disease is a highly heterogeneous disorder. As such, from the available open datasets, the largest sample size that met our inclusion/exclusion criteria was selected. |
| Data exclusions | Exclusionary criteria for all participants included current neurological (other than PD) or psychiatric disorder; MEG contraindications; and unusable MEG, speech sample, or demographic data. |
| Replication | This is not a replication study. However, the general patterns of neurophysiological change seen in our data align well with previous literature. |
| Randomization | Not relevant - participants were assigned based on whether they had a clinical diagnosis of idiopathic Parkinson's disease. |
| Blinding | Blinding during collection was not possible, due to the nature of the open science initiatives for which the data were collected. However, all data collection procedures were standardized. Initial preprocessing was performed on data without group identifiers. |

# Reporting for specific materials, systems and methods

We require information from authors about some types of materials, experimental systems and methods used in many studies. Here, indicate whether each material, system or method listed is relevant to your study. If you are not sure if a list item applies to your research, read the appropriate section before selecting a response.

## Materials & experimental systems

| n/a | Involved in the study |
|---|---|
| ☒ | ☐ Antibodies |
| ☒ | ☐ Eukaryotic cell lines |
| ☒ | ☐ Palaeontology and archaeology |
| ☒ | ☐ Animals and other organisms |
| ☒ | ☐ Clinical data |
| ☒ | ☐ Dual use research of concern |

## Methods

| n/a | Involved in the study |
|---|---|
| ☒ | ☐ ChIP-seq |
| ☒ | ☐ Flow cytometry |
| ☒ | ☐ MRI-based neuroimaging |
